# Protein transduction domain of transactivating transcriptional activator fused to outer membrane protein K of *Vibrio parahaemolyticus* to vaccinate marbled eels (*Anguilla marmorata*) confers protection against mortality caused by *V. parahaemolyticus*

**DOI:** 10.1111/1751-7915.12281

**Published:** 2015-04-27

**Authors:** Hang Wang, Wei Yang, Guoying Shen, Jianting Zhang, Wei Lv, Binfeng Ji, Chun Meng

**Affiliations:** Institute of Pharmaceutical Biotechnology and Engineering, College of Biological Science and Engineering, Fuzhou UniversityFuzhou, Fujian, 350002, China

## Abstract

Although immersion and oral vaccination are the most practical methods for fish farmers, their applications are very limited due to low immune stimulation effect. We used the protein transduction domain (PTD) of transactivating transcriptional factor (TAT) derived from HIV TAT protein to increase the delivery efficiency of aquatic protein vaccines. *Vibrio parahaemolyticus* outer membrane protein K (ompK), a reported vaccine candidate for the prevention of *V. parahaemolyticus* infection, was fused with TAT-PTD expressed in *Escherichia coli*. We found that PTD-ompK fusion protein effectively penetrated into marbled eel bodies. Analysis of ompK antibody titres demonstrated that immersion vaccination with PTD-ompK was superior to ompK alone and induced robust immune stimulation in marbled eels. Both active and passive protection analyses against immersive challenge with *V. parahaemolyticus* strains demonstrated that marbled eels immunized with PTD-ompK survived significantly longer than those immunized with ompK alone. Our results indicated that TAT-PTD could be served as is an efficient delivery system for aquatic immersion vaccinations against various infectious diseases commonly seen in aquatic farm industry.

## Introduction

Fish farming has increased greatly worldwide, infectious fish diseases, such as bacterial, viral, fungal and parasitic infections are still the major constraints to successful expansion of the industry (Pulkkinen *et al*., 2010; Mitchell and Rodger, 2011; Tavornpanich *et al*., 2012; Liu *et al*., 2013; Murray, 2013). Infectious fish diseases have caused massive economic losses in aquaculture. When such a kind of diseases break out, application of large quantity of antibiotics is the main response (Chinabut and Puttinaowarat, 2005; Das *et al*., 2008). Antibiotics in terrestrial and aquatic environment have caught much more social attention worldwide and have emerged as dangerous pollutants hazardous to human health. Antibiotics abuse in aquaculture may become more dangerous than the infectious diseases in fish (Sorum and L'Abee-Lund, 2002; Penders and Stobberingh, 2008; Oliva-Teles, 2012).

Researchers have tested a variety of vaccines to control infectious fish diseases. During the last few years, a few vaccines have been used in commercial aquaculture of Atlantic salmon (*Salmo salar*), rainbow trout (*Salmo gairdneri*) and channel catfish (*Ictalurus punctatus*) (Vandenberg, 2004; Hastein *et al*., 2005; Tafalla *et al*., 2013). In general, the effect of vaccination against bacterial infections was satisfactory. Although immersion and oral vaccines were easy to apply, the best protection was obtained with injection vaccines which were relatively expensive, time consuming and inconvenient for large aquaculture operations. Moreover, injection vaccination caused more adverse reactions in fish (Nakanishi *et al*., 2002; Lin *et al*., 2005; Plant and Lapatra, 2011).

In this study, we developed an immersion vaccine, recombinant fusion protein transduction domain-outer membrane protein (PTD-ompK), in which a transactivating transcriptional factor (TAT)-PTD was introduced to enhance the delivery efficiency of the immunogen for immunization. The efficiency and protective effect of the recombinant PTD-ompK fusion protein was evaluated by challenging marbled eels (*Anguilla marmorata*) with *Vibrio parahaemolyticus* infection.

## Materials and methods

### Reagents, antibodies and plasmid constructs

Culture media, oligonucleotides, protein lysis buffer, isopropylthiogalactoside, deoxyribonucleic acid (DNA) extract kit antibodies, oligonucleotide primers, enhanced chemiluminescence detection solutions and enzymes were purchased from Sango Biotech, China. Antibodies were from Santa Cruz Biotechnology. Virulent *V. parahaemolyticus* was a gift from Dr Hui Chen (Third Institute of Oceanography, China) and used for challenge experiments.

**Fig 1:**
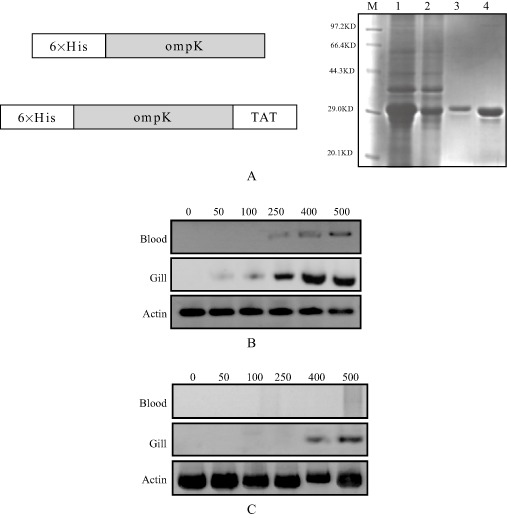
Transactivating transcriptional factor fusion proteins are efficiently transduced into eel organs. A: (left), schematic representation of the ompK constructs used in this study. 6His, 6xhistidine tag; ompK and PTD-ompK constructs were expressed in the *E. coli* BL21 strain and purified as described in the *Materials and methods* section (right). SDS PAGE electrophoresis shows the protein: M, marker protein; 1, cultures containing PTD-ompK; 2, cultures containing ompK; 3, the purified recombinant forms of PTD-ompK; 4, the purified recombinant forms of ompK. Marbled eels were treated with the indicated concentrations of ompK and PTD-ompK for 30 min at 20°C. The gills and blood were then harvested respectively. Gills of 0.5 g lysates washed three times in PBS, and 0.1 ml sera were examined for PTD-ompK (B) and ompK (C) by Western blotting using anti-His antibody. Actin was used as controls.

Protein transduction domain-outer membrane protein K was generated by fusing the TAT-PTD to ompK amino terminal using standard molecular cloning techniques (Fig. 1A). Briefly, the gene encoding ompK was amplified from the genomic DNA extracted from *Vibrio parahaemolyticus* PTD-ompK by polymerase chain reaction (PCR) with ompK primers and PTD-ompK primers contained TAT coding sequences respectively (Table 1). Following digestion of PCR products with restriction enzyme *Bam*HI and *Sal*I, the digested ompK gene fragments were cloned into *Bam*HI and *Sal*I digested plasmid pET28a (Novagen) for ompK-pET28a plasmid construction. *Bam*HI and *Xho*I digested PTD-ompK gene fragments were cloned into *Bam*HI and *Xho*I digested plasmid pET28a for PTD-ompK-pET28a plasmid construction, which *E. coli* DH5 was used as the bacterial host. For recombinant protein expression, the ompK-pET28a and PTD-ompK-pET28a plasmids were transcloned into the *Escherichia coli* expression strain BL21(DE3) respectively.

**Table 1:** The primers used for cloning ompK and PTD-ompK

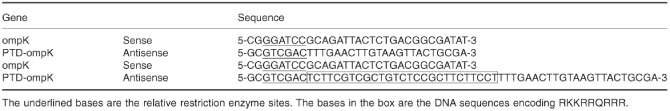

### Protein purification

BL21(DE3) PTD-ompK was grown at 37°C until an OD_600 nm_ of 0.5 was reached. The recombinant protein expression was induced by 0.5 mmol L^−1^ isopropylthiogalactoside treatment for 6 h at 25°C. Bacteria were then harvested and lysed by sonication in 50 ml of denaturing lysis buffer. The His-tagged fusion proteins were purified using Ni-NTA-agarose columns (Sango Biotech) that were pre-equilibrated with denaturing lysis buffer containing 20 mmol L^−1^ of imidazole. Clarified lysates were applied to the columns, and after extensive washing with lysis buffer plus 20 mmol L^−1^ of imidazole, recombinant proteins were eluted with 250 mmol L^−1^ of imidazole according to the manufacturer's instructions. Eluted protein solutions were then dialysed and frozen in 10% glycerol at −80°C.

### Western blot analysis

For preparation of tissue protein extracts, 0.5 g tissue was ground in liquid nitrogen, and then 2 ml ice-cold lysis buffer was added for 15 min in ice. Lysates were clarified by centrifugation at 15 000× g for 15 min at 4°C. Protein extracts (30 μg per lane) were separated by SDS-PAGE and transferred to a polyvinylidene difluoride membrane. Membranes were probed by immunoblotting with primary and horseradish peroxidase (HRP)-conjugated secondary antibodies followed by ECL detection as described before (McManus *et al*., 2009).

### Enzyme-linked immunosorbent assay (ELISA)

To determine specific serum antibody titres, 96-well polystyrene microtitre plates were activated with 2 g ml^−1^ of poly-L-lysine in carbonate buffer, pH 9.6, at 37°C for 2 h. Plates were washed three times with double distilled water. Then they were coated with 10 mg L^−1^ sample diluted in PBS, pH 7.6, at 4°C overnight. The plates were re-washed three times with PBS contained 0.05% (v : v) Tween 20 (T-PBS), and were blocked with 1% skim milk in T-PBS. Twofold serum dilutions were prepared in T-PBS and incubated at 37°C for 2 h in the plates. After three washes with T-PBS, the plates were incubated with anti-mouse IgG-HRP for 2 h at room temperature. Finally, the plates were washed as indicated, and the substrate H_2_O_2_ (3%) and o-phenylenediamine (0.5 mg ml^−1^) in citrate buffer 0.1 M (pH 5) was used to develop the chromogenic reaction for 15 min (Guirola *et al*., 2006).

### Immunization of marbled eels with vaccines and antisera specific to ompK

Marbled eels, whose sizes were 0.1 to 0.3 g in body weight (6–8 cm in body length) were purchased from Guangdong Yuqiangfeng hatchery. They were allowed to adapt for 1 week in aerated water at 20°C. The health status of the fish was examined according to the methods (Burzynska and Maciejska, 1974). The fish were then randomly divided into groups and kept in 50 L tanks in aerated water at 30°C (half of the water was changed daily during experimental period) and fed with commercial pellets. In this work, we adopted lethal dose of *V. parahaemolyticus* to infect marbled eels as challenge evaluation of ompK vaccine, which is usually used in efficiency evaluation of other vaccines (Dolby and Standfast, 1958; Yoshida *et al*., 1970; Paoletti *et al*., 1994; Leary *et al*., 1995).

The ompK or PTD-ompK vaccine was administered using the following procedures: (i) groups of marbled eels were vaccinated by dip (30 min) in the required dilution (the final concentration was 0.1 mg ml^−1^) of the vaccine in aerated sea water, and (ii) groups of marbled eels were vaccinated by i.p. injection of 0.1 ml of the vaccine. Groups of marbled eels which were not vaccinated were used as controls. All experiments were performed in duplicate (the second vaccination was performed after 30 days of the first vaccination). According to the reported work, efficient immune response might produce at about 30 days (Austin, 1984). So we strengthen the immune reaction through second vaccination at the 30th day after the primary vaccination. At 7 days after the second immunization, each group of eels was challenged with a lethal dosage of virulent *V. parahaemolyticus* suspended in sterile PBS through 20 min immersion, and the survival time of each eel was monitored for 14 days.

Antisera-mediated passive protection assays were performed as previously described as performing on mice (Green *et al*., 2005). Briefly, groups of 10 marbled eels each were passively immunized with 100 ul of each of the antiserum with i.p. injection. Control marbled eels were injected i.p. with 100 ul of pooled serum from placebo immunized marbled eels. At 24 h after the immunization, each group of eels was challenged with a lethal dosage of virulent *V. parahaemolyticus* suspended in sterile PBS through 20 min immersion, and the survival time of each eel was monitored for 14 days.

### Statistical analysis

All experiments were repeated at least three times. Conventional statistical methods were used to calculate means ± SE Student's *t*-test was used to compare differential groups. A value of *P* < 0.05 was considered statistically significant.

## Results

### PTD-ompK fusion proteins could be efficiently transduced into the gills and blood of marbled eels

His-PTD-ompK was constructed by fusing the nine amino acids of the TAT-PTD to the amino terminus of the full-length ompK. Both recombinant His-TAT-ompK and His-ompK proteins were produced following bacterial expression and purification using Ni-NTA-agarose columns respectively. The results analysed by SDS-PAGE demonstrated that the molecular sizes were consistent with the predicted molecular weights based on their sequences (Fig. 1A, right).

The marbled eels were immersed with increasing amount of PTD-ompK or ompK respectively. Western blotting result showed that PTD-ompK protein entered the gills of eels from 50 to 500 nmol L^−1^ treatment and entered the blood from 250 to 500 nmol L^−1^ treatment in dose-dependent manners (Fig. 1B). Whereas, ompK could be found in the gills at 400 nmol L^−1^ ompK. Outer membrane protein K was undetectable in the blood of eels even with 500 nmol L^−1^ immersion treatment (Fig. 1C). For the following experiments, we selected 500 nmol L^−1^ protein as the working concentration.

### Comparison of antibody levels specific to ompK when vaccinized with different delivery methods

**Fig 2:**
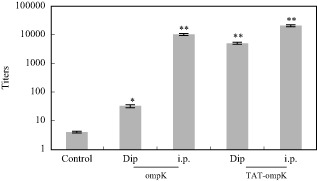
Specific antibody titres detected by ELISA in the sera of fish vaccinated with different ways.Dip: Dip immersion vaccination; i.p.: intraperitoneal injection vaccination. *P < 0.05; **P < 0.001.

To assess whether PTD-ompK fusion proteins could enhance the immune-stimulatory effects, we first treated eels with 500 nmol L^−1^ PTD-ompK or ompK protein with dip immersion and intraperitoneal injection respectively, and antibodies specific to ompK were determined by enzyme-linked immunosorbent assay (ELISA) after twice vaccination. Intraperitoneal injection for both PTD-ompK and ompK exhibited good immune-stimulatory effect. The titer of antibody in secondary response serum to ompK was higher by 1000-fold in ompK-vaccinated group and 2000-fold in PTD-ompK group than that in control group (Fig. 2).

We found that PTD-ompK, but not ompK, induced a high level antibody specific to ompK in eel blood when eels were vaccinated with dip immersion. The titres of secondary response serum reached 1:5120 in PTD-ompK groups, only 1:32 in ompK groups. Although the titre induced with intraperitoneal injection was significantly higher than with dip immersion vaccination, the TAT-PTD could efficiently deliver macromolecular ompK into ell bodies and stimulate high levels of specific antibody production (Fig. 2). Therefore, TAT-PTD could significantly enhance immersion vaccination efficiency.

### Protection of marbled eels from lethal systemic infection with *V. parahaemolyticus*

**Fig 3:**
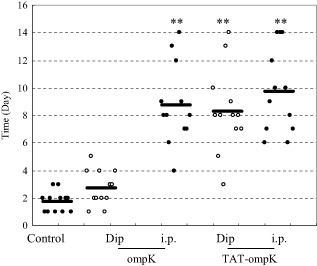
Survival times for marbled eels after *V. parahaemolyticus* challenge. Groups of 12 marbled eels were immunized with the indicated antigens and were challenged with approximately 2.53 × 10^7^ cfu ml^−1^ *V. parahaemolyticus* after 2 weeks of the second immunization. Each datum point represents one eel. A horizontal line denotes the median survival time for the group. **P < 0.001. The other two experiments were shown in Fig. S1.

To investigate whether TAT-PTD mediated vaccination could efficiently protect against *V. parahaemolyticus*, four groups of marbled eels were immunized twice with 500 nmol L^−1^ ompK or PTD-ompK by dip immersion and intraperitoneal injection respectively. Each group was challenged with *V. parahaemolyticus* at the 10th day after the second immunization. Compared with the control group immunized with normal saline, each of the treatment groups elicited different protection against *V. parahaemolyticus*. Compared with the control group, ompK with dip immersion (*P* > 0.05), PTD-ompK with dip immersion (*P* < 0.001), ompK with intraperitoneal injection (*P* < 0.001) and PTD-ompK with intraperitoneal injection (*P* < 0.001) elicited different protection (Fig. 3).

Marbled eels immunized with PTD-ompK survived significantly longer (about 5 days) than those with ompK alone in dip immersion vaccination. Furthermore, eels immunized with PTD-ompK survived a little longer than those immunized with ompK even through injection vaccination ways. Overall, our data supported that PTD-ompK fusion protein produced satisfactory protection, superior to ompK alone, against *V. parahaemolyticus* by dip immersion vaccination.

### Passive protection by immunization of ompK-specific antisera

To examine whether the protection was mediated by specific antibodies to opmK, marbled eels were passively immunized by peritoneal injection with the sera from the marbled eels previously immunized by dip immersion or peritoneal injection with opmK, TAT-opmK or normal saline respectively. Each treatment group was challenged with lethal dosages of live *V. parahaemolyticus*.

**Fig 4:**
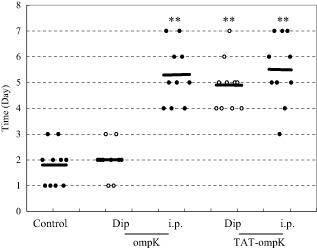
Survival times for eels after challenge. Groups of 10 marbled eels were immunized with the indicated sera containing specific polyclonal antisera.Challenged was performed with approximately 2.53 × 10^7^ cfu ml^−1^ *V. parahaemolyticu*. Each datum point represents one eel. A horizontal line denotes the median survival time for the group. **P < 0.001. The other two experiments were shown in Fig. S2.

As seen with active immunization, marbled eels that received different antisera-containing specific antibodies to opmK showed different response to the tack of *V. parahaemolyticus*. Antisera from eels vaccinated with PTD-ompK by dip immersion exhibited strong protection against *V*. *parahaemolyticus*, and eels so immunized survived significantly longer than those with ompK alone by dip immersion, which survived slightly longer (2 days) than those in the control group (*P* > 0.1) (Fig. 4).

Our data demonstrated that anti-TAT-opmK sera of both dip immersion and peritoneal injection produced longer survival time than the sera of ompK alone group. Furthermore, PTD-ompK fusion protein strategy in immersion vaccination could generate strong immune-stimulatory effect comparable to that obtained by route of injection and is warranted for further development as an important delivery system of dip-immersion vaccination against *V. parahaemolyticus* or other infectious pathogens in aquaculture.

## Discussion

It was reported that TAT-PTD could efficiently cross biological membranes and facilitate the delivery of macromolecular proteins into various cell types (Wadia and Dowdy, 2003; Gump and Dowdy, 2007). Therefore, we hypothesized that proteins fused with TAT-PTD could be a rational solution for the low efficiency of immersion vaccination.

The outer membrane proteins of the fish pathogen, *V. parahaemolyticus*, which play important roles in the interaction between bacteria and hosts, are potential candidates for the development of vaccines. It was reported that the outer membrane protein, ompK could elicit immune protection in fish species against *V. parahaemolyticus* infections (Ningqiu *et al*., 2008; Li *et al*., 2010; 2013). However, the inconvenience of vaccination application hindered its successful development as an effective vaccine against the deadly infection by *V. parahaemolyticus* in fish (Collado *et al*., 2000; Romoren *et al*., 2002; Clark and Cassidy-Hanley, 2005; Companjen *et al*., 2006; Plant and Lapatra, 2011; Fredriksen and Grip, 2012; Ruyra *et al*., 2013; Vimal *et al*., 2013).

The most critical factor has been the lack of effective methods in aquaculture. Although oral and immersion vaccinations are the most favorable and least stressful strategies for fish, they are not as effective as injection vaccination, which is by far the most efficient route for vaccination in terms of duration and strength of immune protection generated. However, injection vaccination is neither practical nor economical for large-scale vaccination in aquaculture.

The TAT-PTD was reported to successfully transduce bioactive proteins into mammalian cells *in vivo* (Wadia and Dowdy, 2003; Gump and Dowdy, 2007). We developed a fusion protein by fusing PTD to ompK, an outer membrane protein of *V. parahaemolyticus*, an effective and easy-to-administer vaccine against *V. parahaemolyticus* infection in fish and demonstrated the efficacy of the new vaccination by dip immersion in comparison with the injection immunization.

Western blot data clearly demonstrated that PTD-ompK, not ompK, was able to enter the gills and the blood of the marbled eels in dose-dependent manners when marbled eels were immunized with immersion method. The followed ELISA analyses further verified that strong immune responses with ompK-specific antibodies were generated in marbled eels after immunization with PTD-ompK either by dip immersion or by peritoneal injection.

To further demonstrate the immune protection and survival benefits of the developed vaccine with PTD-ompK fusion protein, a lethal dose of live *V. parahaemolyticus*, which killed rapidly the non-immunized marbled eels within 3 to 4 days, was applied to the various vaccinated groups. In the active immunization test, marbled eels immunized with PTD-ompK survived significantly longer than those immunized with ompK alone by either dip immersion or by peritoneal injection. Although PTD-ompK group by dip immersion elicited slightly less protection than by intraperitoneal injection, most marbled eels in this group survived much longer than those with ompK alone.

In the passive immunization test, we obtained similar protective efficacy for each of the immunized groups. Marbled eels immunized with the sera from the previously PTD-ompK immunized eels survived significantly longer than those with ompK alone. In summary, our data demonstrated that PTD-ompK fusion protein facilitated the immunogen getting into the bodies of the tested marbled eels and generated immune responses *in vivo* by dip immersion, which was stronger than imunogen alone and comparable to that obtained by injection immunization method. It is clear that the environment affects the immune system of fish (Martins *et al*., 2011). We have not studied the effect of environmental variations on immune responses of PTD-ompK vaccine in this work. We will examine the changes of environmental factors on immune efficacy and optimize the vaccination strategy in the future work.

This study has provided strong evidence that PTD-ompK fusion protein enhances the vaccination efficiency of dip-immersion method for immunizing marbled eels against the infection by *V. parahaemolyticus* and PTD fusion protein as a useful strategy for developing better vaccines against bacterial infections or infections by other pathogens such as virus or fungi commonly seen in aquaculture. The success of dip-immersion vaccination with PTD-ompK fusion protein may further facilitate the large-scale application of vaccinations in fish farm industry.

## Supporting Information

Fig S1 Survival times for marble eels after *V. parahaemolyticus* challenge. Two groups of 10 marble eels were immunized with the indicated antigens and were challenged with approximately 2.53 × 10^7^ cfu ml^−1^ *V. parahaemolyticus* after 2 weeks of the second immunization. Each datum point represents one eel. A horizontal line denotes the median survival time for the group. ***P* < 0.001.

Fig S2 Survival times for eels after challenge. Two groups of 10 marble eels were immunized with the indicated sera containing specific polyclonal antisera. Challenged was performed with approximately 2.53 × 10^7^ cfu ml^−1^ *V. parahaemolyticus*. Each datum point represents one eel. A horizontal line denotes the median survival time for the group. * *P* < 0.05; ** *P* < 0.01.
