# Supplementary material for: Protein transduction domain of transactivating transcriptional activator fused to outer membrane protein K of Vibrio parahaemolyticus to vaccinate marbled eels (Anguilla marmorata) confers protection against mortality caused by V. parahaemolyticus
Source: Microb Biotechnol. 2015 Apr 27;8(4):673–80. doi: 10.1111/1751-7915.12281 (PMC4476822; doi:10.1111/1751-7915.12281)
Supplement: Fig S1 — Survival times for marble eels after V. parahaemolyticus challenge. Two groups of 10 marble eels were immunized with the indicated antigens and were challenged with approximately 2.53 × 10^7^ cfu ml−1 V. parahaemolyticus after 2 weeks of the second immunization. Each datum point represents one eel. A horizontal line denotes the median survival time for the group. **P < 0.001. [file mbt20008-0673-sd1.docx]

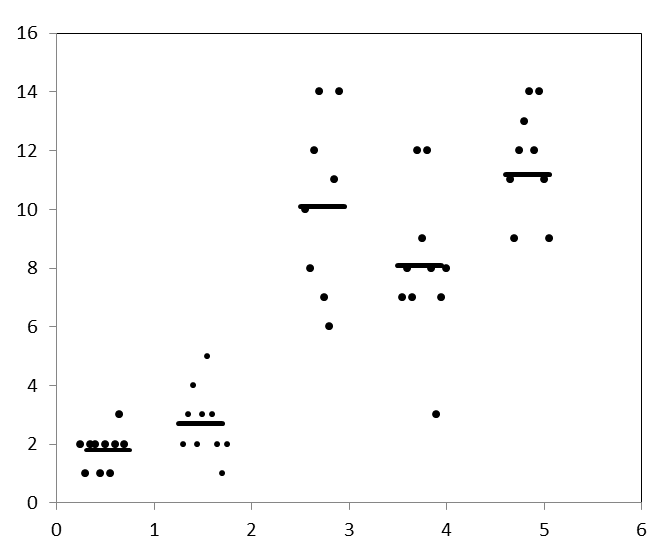


**

**

**

Control Dip i.p. Dip i.p.

TAT-ompK

ompK

A

**

**

**

Control Dip i.p. Dip i.p.

TAT-ompK

ompK

B

Figure S1 Survival times for Marble eels after *Vibrio parahaemolyticus* challenge. Two groups of 10 Marble eels were immunized with the indicated antigens and were challenged with approximately 2.53×10^7^ cfu/mL *Vibrio parahaemolyticus* after two weeks of the second immunization. Each datum point represents one ell. A horizontal line denotes the median survival time for the group. ** p<0.001.
