# Supplementary material for: Protein transduction domain of transactivating transcriptional activator fused to outer membrane protein K of Vibrio parahaemolyticus to vaccinate marbled eels (Anguilla marmorata) confers protection against mortality caused by V. parahaemolyticus
Source: Microb Biotechnol. 2015 Apr 27;8(4):673–80. doi: 10.1111/1751-7915.12281 (PMC4476822; doi:10.1111/1751-7915.12281)
Supplement: Fig S2 — Survival times for eels after challenge. Two groups of 10 marble eels were immunized with the indicated sera containing specific polyclonal antisera. Challenged was performed with approximately 2.53 × 10^7^ cfu ml−1 V. parahaemolyticus. Each datum point represents one eel. A horizontal line denotes the median survival time for the group. * P < 0.05; ** P < 0.01. [file mbt20008-0673-sd2.docx]

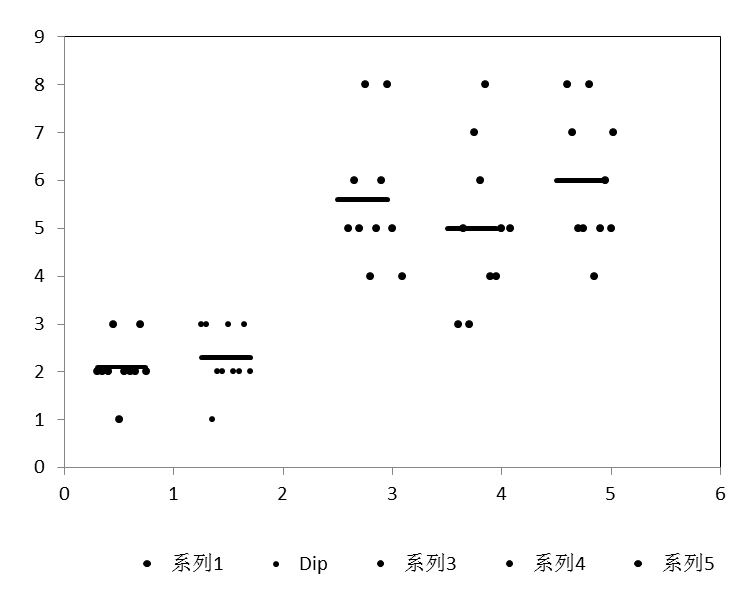


**

*

**

Control Dip i.p. Dip i.p.

TAT-ompK

ompK


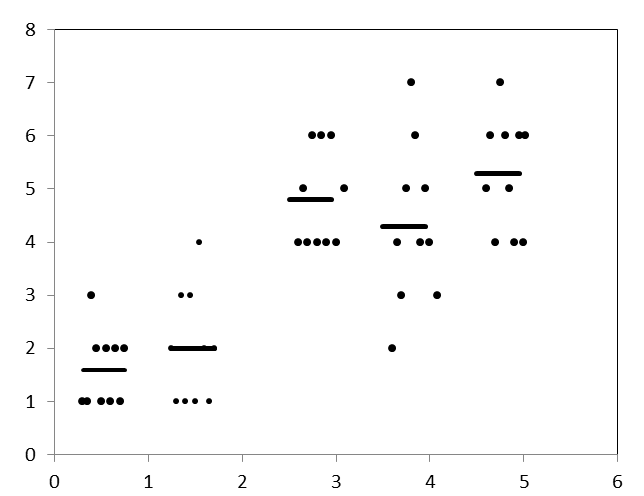


*

**

**

Control Dip i.p. Dip i.p.

TAT-ompK

ompK

Figure S2 Survival times for eels after challenge. Two groups of 10 Marble eels were immunized with the indicated sera containing specific polyclonal antisera. Challenged was performed with approximately approximately 2.53×10^7^ cfu/mL *Vibrio parahaemolyticu*. Each datum point represents one eel. A horizontal line denotes the median survival time for the group. * p<0.05; ** p<0.01.
